# 367. Enhancing antimicrobial stewardship capacity building and communication across multidisciplinary departments in Chinese hospitals: a national qualitative study

**DOI:** 10.1093/ofid/ofae631.108

**Published:** 2025-01-29

**Authors:** Xiaomin Wang, Xudong Zhou

**Affiliations:** Hangzhou Normal University, Hangzhou, Zhejiang, China (People's Republic); Zhejiang University, Hangzhou, Zhejiang, China (People's Republic)

## Abstract

**Background:**

Antimicrobial stewardship (AMS) programs have been widely adopted and proven to be effective to optimize antibiotic use. After a decade of efforts in mandatory AMS implementation, inappropriate prescribing and antimicrobial resistance are still disproportionately prevalent in China. We aim to investigated the underlying barriers to the mandatory AMS programs from a multidisciplinary health system perspective in the Chinese context.

Example of quotes from interview transcripts that illustrate and validate the themes

**Figure:**
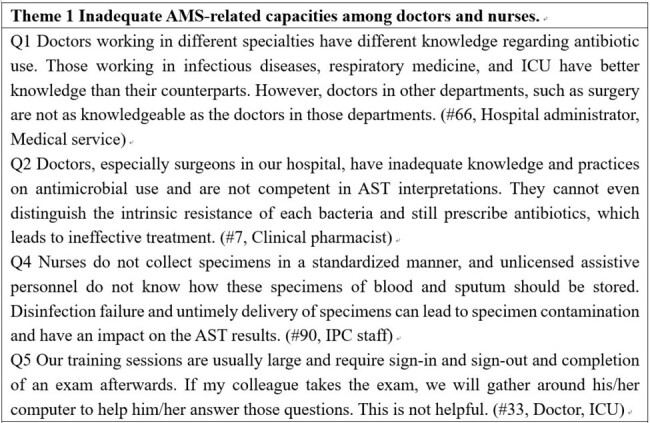

**Methods:**

Face-to-face, in-depth qualitative interviews were conducted in 16 public hospitals in eastern, central, and western China. Hospitals were purposely selected with full consideration to represent both economically developing and developed areas and both secondary and tertiary care hospitals. A total of 111 respondents were interviewed, including 38 doctors, 28 clinical pharmacists, 15 microbiologists, 14 infection prevention and control (IPC) specialists, 10 experts from medical service departments, and 6 quality improvement experts. A thematic framework analysis was conducted aided by NVIVO 11 software.

Example of quotes from interview transcripts that illustrate and validate the themes (continue)

**Figure:**
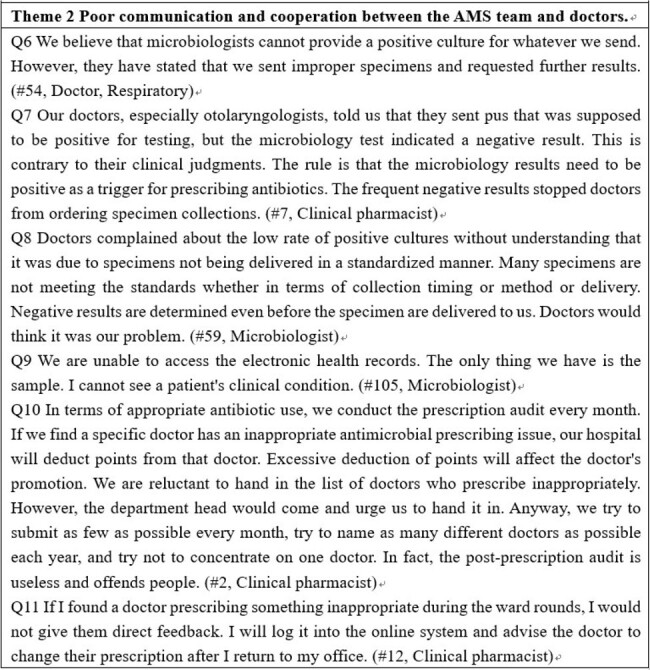

**Results:**

Our research identified two key barriers to AMS implementation. 1) Inadequate AMS-related capacities among doctors and nurses. Doctors, especially surgeons, had inadequate knowledge and poor practices on appropriate antibiotic use and had insufficient knowledge and skills in antimicrobial susceptibility testing (AST) and its interpretation. The continuing education in a few hospitals was reported to be crammed with large class sizes and showed little effect on capacity improvement. 2) Poor communication and cooperation between the AMS team and doctors. Compared with AMS team members, doctors had a dominant and powerful position in hospitals. Clinical pharmacists admitted that they were subjected to high pressure and not well respected while conducting routine prescription audit and feedback works. Most (87%) of the interviewed microbiologists were not able to access the electronic health records.

**Conclusion:**

We propose a multi-disciplinary training program covering microbiology, clinical pharmacy, and IPC for newly-recruited health workers (i.e., doctors and nurses), and a communication mechanism among clinical departments and AMS members to facilitate AMS implementation.

**Disclosures:**

**All Authors**: No reported disclosures

